# Vertebral Artery Dissection and Cord Infarction - an Uncommon Cause of Brown-Séquard and Horner Syndromes

**DOI:** 10.7759/cureus.308

**Published:** 2015-08-21

**Authors:** Jason Ginos, Scott Mcnally, Melissa Cortez, Edward Quigley, Lubdha M Shah

**Affiliations:** 1 Department of Radiology, University of Utah; 2 Department of Neurology, University of Utah; 3 Huntsman Cancer Institute, University of Utah

**Keywords:** vertebral artery dissection, horner syndrome, brown-sequard, cord infarction

## Abstract

This case report illustrates the neuroanatomy and neurovascular anatomy of the cervical spinal cord by exploring the pathophysiology of cervical cord infarction secondary to vertebral artery injury.

The spinal cord is made up of several important tracts, including the dorsal column medial lemniscus system, corticospinal tracts, and the anterolateral system. Injury to one or more of these pathways can result in localizing neurological symptoms. Also contributing to the complexity of spinal vascular pathophysiology is the considerable variation to the cervical cord vascular anatomy.

Understanding spinal cord function and neuroanatomy can aid in prompt diagnosis and management of ischemic cord lesions. In combination with a thorough clinical exam, advanced imaging techniques, such as diffusion tensor imaging, can not only localize the injury but also potentially help predict functional outcome.

## Introduction

The imaging and clinical manifestations of the pathologic processes affecting the cervical spinal cord can be challenging to elucidate. This case illustrates the complex neurovascular anatomy and neuroanatomy, the understanding of which is essential for prompt diagnosis and management of ischemic cord lesions. Magnetic resonance imaging provides structural information to help localize the site of injury and potential causes. Advanced imaging techniques, such as diffusion tensor imaging, may potentially help predict functional outcome.

## Case presentation

### History

A 33-year-old female presented to the emergency department after developing acute left-hand numbness and left-sided weakness after neck manipulation. On physical exam, she was found to have a left-sided ptosis and miosis and right-sided hyperesthesia below the mid-thoracic level. Informed patient consent was obtained for treatment. After hospital admission, the patient underwent a full but unrevealing neurologic workup. The patient was discharged with the diagnosis of conversion disorder, sent home with a cane, and prescribed aspirin.

At the two-week follow-up neurology appointment, she had a persistent left Horner syndrome and left leg weakness (4/5) as well as the loss of vibratory sensation and proprioception on the left and altered pain sensation on the right below the cervical level.

### Imaging findings

**Figure 1:**
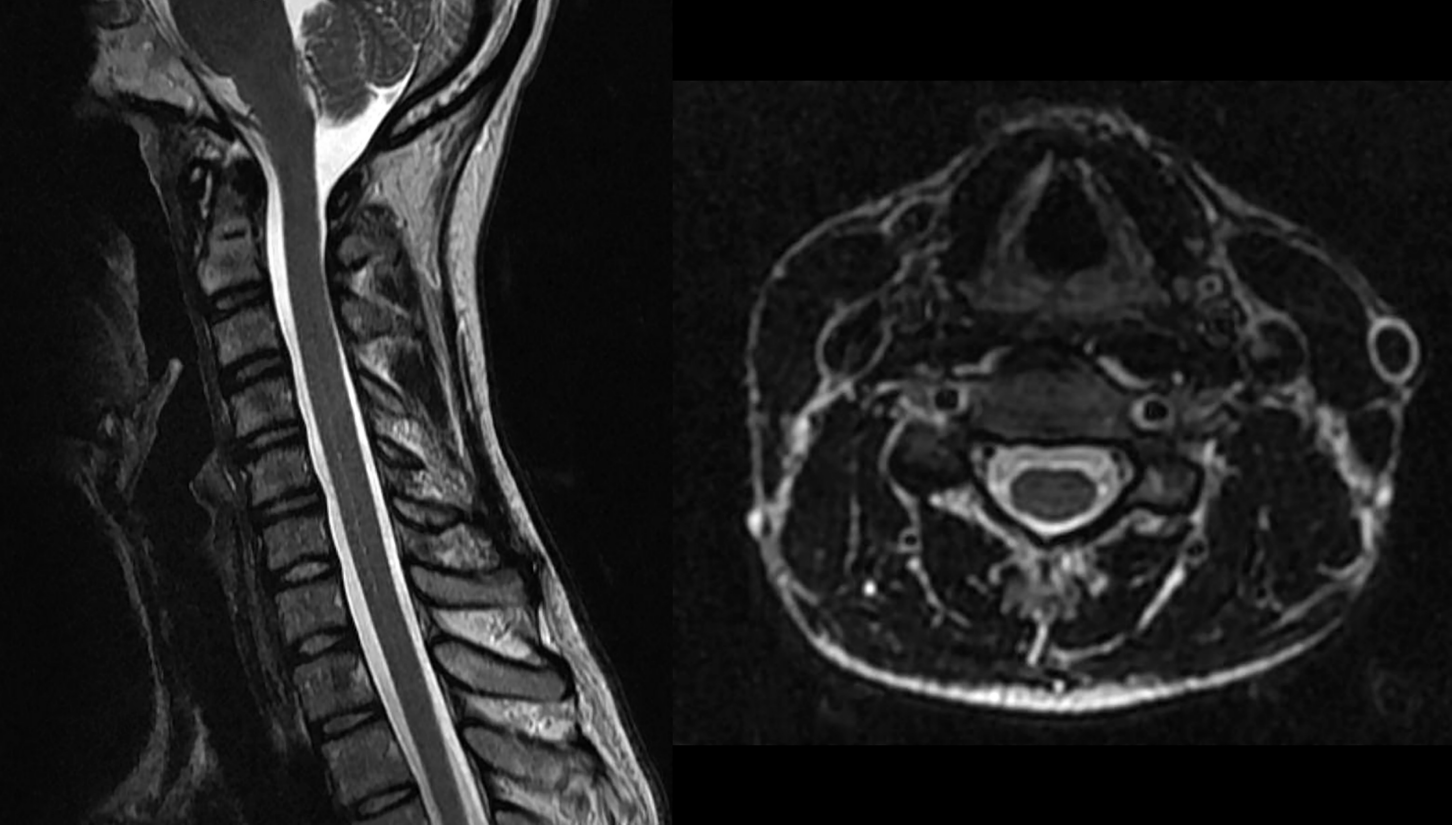
Sagittal and axial T2-weighted images demonstrate normal cervical cord signal intensity and morphology Note that bilateral vertebral artery flow voids are normal.

**Figure 2:**
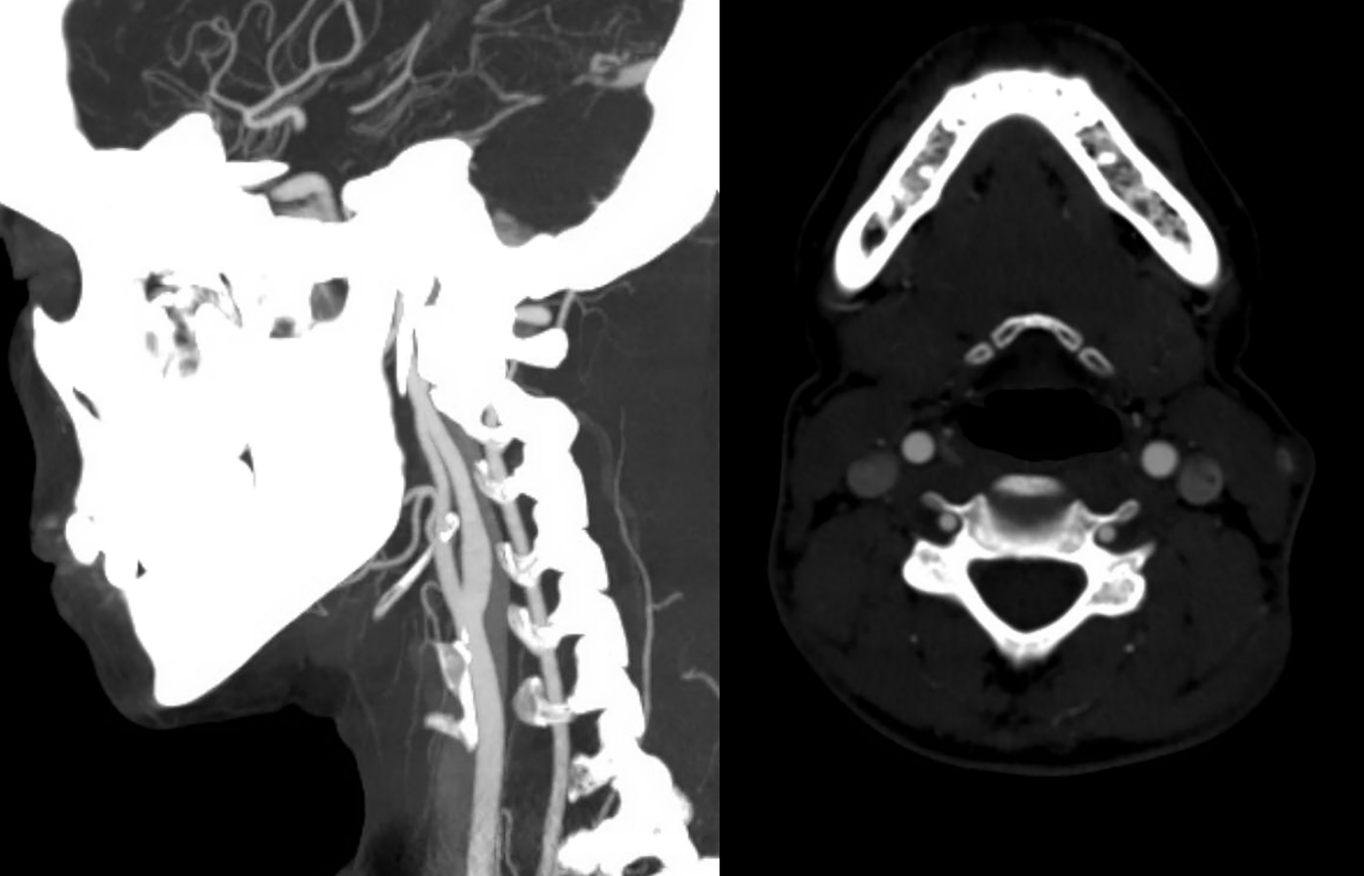
Sagittal and axial CT angiogram images demonstrate normal vertebral arteries without evidence of dissection

Initial brain magnetic resonance imaging (MRI), complete spine MRI (Figure 1), and cervical computed tomography angiogram (CTA) (Figure 2) were negative. 

**Figure 3:**
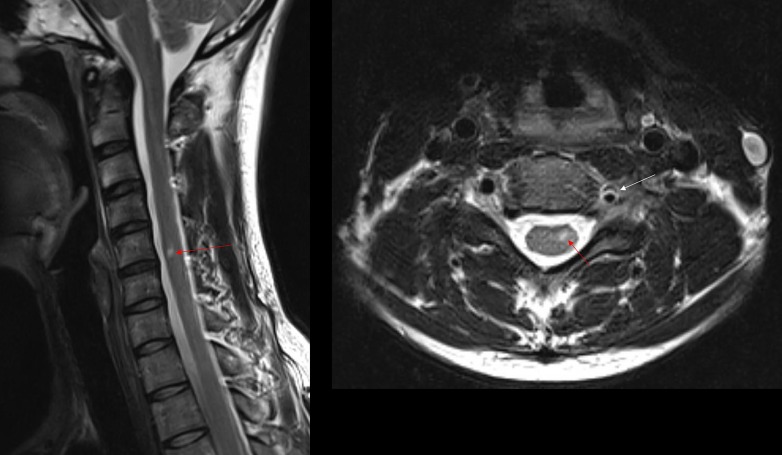
Sagittal and axial T2-weighted images demonstrate hyperintensity within the left hemicord at the C5 level (red arrow) Note that the left vertebral artery flow void is irregular and narrowed (white arrow).

**Figure 4:**
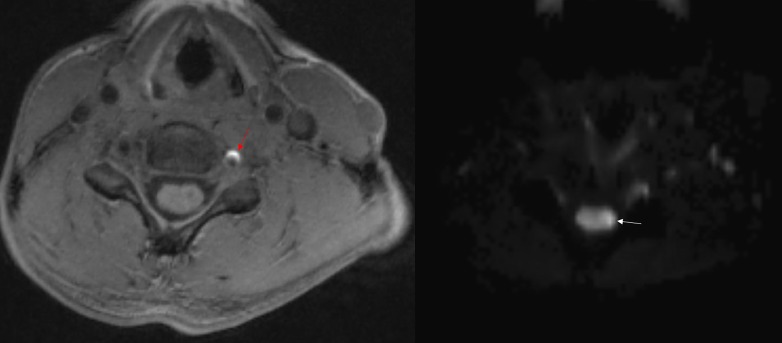
Axial T1-weighted (MPRAGE) image with fat saturation demonstrates left vertebral artery dissection with luminal narrowing and hyperintense intramural hematoma (red arrow) Axial diffusion weighted image (DTI trace) at the same level demonstrates diffusion restriction (white arrow).

The follow-up cervical spine MRI (two weeks later) demonstrated diffusion restriction and T2 hyperintensity involving the left hemicord at the C5 level in keeping with an acute infarction (Figure 3). In addition, the adjacent left vertebral artery was dissected with luminal narrowing and intramural hematoma (Figure 4).

## Discussion

There is a considerable variation to the cervical cord vascular anatomy; however, knowledge of the fundamental tracts and vascular supply can aid in understanding the spinal pathophysiology. Arterial supply to the cervical cord includes branches of the vertebral arteries, the ascending cervical arteries, and occasionally the ascending pharyngeal and occipital arteries. The anterior spinal artery and two posterior spinal arteries constitute the majority of the vascular supply to the cervical cord with contributions from the anterior radiculomedullary arteries [1].

Spontaneous, traumatic, or iatrogenic injury to these vessels can result in lateral cord infarction, leading to a variety of complex neurologic syndromes.

**Figure 5:**
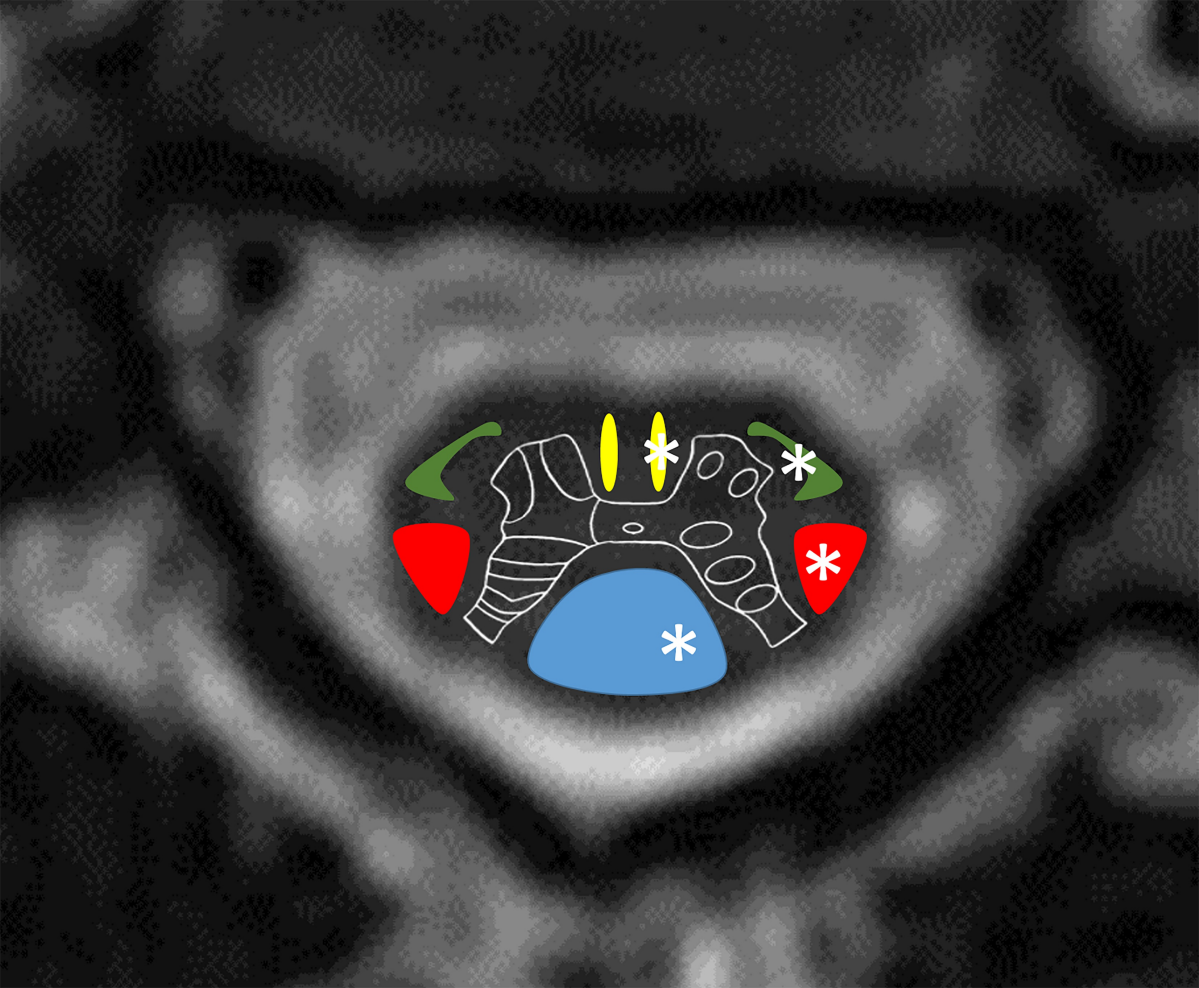
Cross section graphic demonstrates the cervical cord anatomy Dorsal column-medial lemniscus system (blue); corticospinal tracts: lateral corticospinal tract (red) and anterior corticospinal tract (yellow); anterolateral system (green). Injury in this case involved the left hemicord damaging each of these pathways (*).

The Brown-Séquard syndrome is defined as an ipsilateral loss of proprioception, vibration, and motor function below the level of the spinal cord injury and contralateral loss of pain and temperature sensation [2]. This pattern of deficits occurs as a result of a direct injury to the ipsilateral dorsal column-medial lemniscus tracts (sensory) and the lateral corticospinal tract (motor). The contralateral loss of pain and temperature sensation occurs via damage to the spinothalamic tract of the anterolateral system (Figure 5). Decussation of these fibers within the cord results in symptoms on the contralateral side. If there is a coexistent injury to the ipsilateral sympathetic pathway, this results in Horner syndrome (ptosis, miosis, and anhidrosis).

Our review of the literature failed to find similar cases of delayed imaging findings of vertebral artery dissection and cord infarct, although these may be underreported. Early recognition of localizing clinical features and imaging findings that are suspicious for vertebral artery dissection are important, as treatment is paramount to reducing morbidity. This case demonstrates that imaging findings of a vertebral artery dissection can be delayed, specifically in the setting of an associated cord infarction. In such cases where clinical suspicion for dissection is high, empiric treatment should begin immediately and close follow-up imaging may be indicated. 

In the literature, CTA is reportedly 100% sensitive and 98% specific in detecting vertebral artery dissection compared to digital subtraction angiography (DSA); however, false positives and negatives are likely underreported, given inherent pitfalls and difficulties in interpreting these imaging studies [3-4]. Even after retrospective review, the initial imaging in our case was unconvincing for definite abnormality.

Cervical cord infarction can manifest in various ways and is an unusual sequela of vertebral artery dissection [5]. Our patient exhibited left-sided weakness and split sensory changes below the mid-thoracic level consistent with Brown-Séquard syndrome as well as left-sided (ipsilateral) ptosis and miosis consistent with an associated Horner syndrome. In this case, we postulate that the left vertebral artery dissection led to occlusion of a radicular branch, resulting in associated cervical cord infarction at the C5 level. The symptoms were due to the involvement of the ipsilateral dorsal column-medial lemniscus tracts (sensory), the lateral corticospinal tract (motor), the contralateral spinothalamic tract, and ipsilateral sympathetic pathway.

## Conclusions

In summary, we present a case that illustrates potential pitfalls in the diagnosis of cervical cord infarction associated with vertebral dissection, including variable neurovascular anatomy and possible delayed imaging manifestations in the acute phase of the clinical presentation. Understanding structural and vascular spinal cord neuroanatomy can aid in prompt diagnosis and management of traumatic and ischemic cord lesions.
